# Analysis of the profile of patients admitted to an orofacial cleft rehabilitation service: An observational study, Cuiabá, 2005-2023

**DOI:** 10.1590/S2237-96222025v34e20240926.en

**Published:** 2025-10-24

**Authors:** Brunna Almeida Guerreiro, Lorraynne dos Santos Lara, Ivan Onone Gialain, Carolina Silvano Vilarinho da Silva, Luiz Evaristo Ricci Volpato

**Affiliations:** 1 Universidade de Cuiabá, Programa de Pós-graduação em Ciências Odontológicas Integradas, Cuiabá, MT, Brazil; 2 Hospital Geral de Cuiabá, Departamento de Odontologia, Cuiabá, MT, Brazil; 3 Hospital de Câncer de Mato Grosso, Departamento de Odontologia, Cuiabá, MT, Brazil

**Keywords:** Epidemiology, Patient Care Team, Cleft Lip, Cleft Palate, Public Health Dentistry, Epidemiología, Grupo de Atención al Paciente, Labio Leporino, Fisura del Paladar, Odontología en Salud Pública

## Abstract

**Objective:**

To analyze the profile of individuals with nonsyndromic orofacial clefts from a reference service in Brazil.

**Methods:**

This retrospective observational study with patients admitted to the Cleft Lip and Palate Rehabilitation Service at the General Hospital of Cuiabá, Mato Grosso, between 2005 and 2023, considered age group, sex, type of cleft, city and area of residence, history of surgeries, and year of admission. Data were obtained from clinical records. Descriptive analysis of the variables was performed, with absolute and relative frequencies, and binomial logistic regression, considering age <3 or ≥3 years old, gender, type of cleft, city and area of residence, with confidence intervals of 95%.

**Results:**

Of the 1,417 patients analyzed, 55.7% were male, 48.2% had cleft lip and palate, 90.0% were from urban areas, 64.1% were from cities in the inland of Mato Grosso, and 57.5% had no history of previous surgeries. The profile of the patients underwent significant changes in the period studied. In the first years, individuals aged ≥3 years old (68.5%) and those with previous surgeries (61.8%) prevailed, while in the later periods, individuals under 3 years old (72.3%) and those who had no surgeries (73.2%) prevailed.

**Conclusion:**

The patient profile reveals a predominance of male individuals with cleft lip and palate from inland cities in Mato Grosso, residing in urban areas, and with no history of surgeries. This profile has evolved from a larger admission of older patients with prior surgery in the first years to younger patients without prior surgery in the most recent years analyzed*.*

## Resumen

**Objetivo:**

: Analizar el perfil de individuos con fisuras orofaciales no sindrómicas atendidos en un servicio de referencia en Brasil.

**Métodos:**

: Este estudio observacional retrospectivo incluyó a pacientes ingresantes en el Servicio de Rehabilitación de Fisuras Labiopalatinas del Hospital General de Cuiabá, Mato Grosso, entre 2005 y 2023. Se consideraron variables como rango etario, sexo, tipo de fisura, municipio de residencia, zona de procedencia, antecedentes quirúrgicos y año de ingreso. Los datos se obtuvieron a partir de los registros clínicos. Se realizó un análisis descriptivo de las variables, con frecuencias absolutas y relativas, y una regresión logística binomial considerando rango etario (<3 o ≥3 años de edad) y sexo, tipo de fisura, municipio de origen y zona de procedencia, con intervalos de confianza del 95 %.

**Resultados:**

: De los 1.417 pacientes analizados, el 55,7 % era de sexo masculino, el 48,2 % presentaba fisura labiopalatina, el 90,0 % provenía de zonas urbanas, el 64,1 % de municipios del interior de Mato Grosso y el 57,5 % no tenía antecedentes de cirugía previa. El perfil de los pacientes mostró cambios significativos durante el período estudiado. En los primeros años, predominaban individuos de ≥3 años de edad (68,5 %) y con cirugías previas (61,8 %), mientras que en los últimos períodos predominaban los menores de 3 años (72,3 %) y sin cirugías previas (73,2 %).

**Conclusión:**

: El perfil de los pacientes revela un predominio de individuos de sexo masculino, con fisura labiopalatina, procedentes de municipios del interior de Mato Grosso, residentes en zonas urbanas y sin antecedentes quirúrgicos. Este perfil cambió a lo largo del tiempo, pasando de un ingreso mayor de pacientes de mayor edad y con cirugía previa en los primeros años, a pacientes más jóvenes y sin cirugía previa en los últimos años analizados*.*

**Ethical aspects**

This research respected ethical principles, having obtained the following approval data:

Research Ethics Committee: Universidade de Cuiabá

Opinion number: 3,610,573

Approval date: 30/9/2019

Certificate of Submission for Ethical Appraisal: 21617419.9.0000.5165

Informed Consent Form: Obtained from all participants before data collection.

## Introduction

Orofacial clefts are the most common congenital anomalies affecting humans, originating from failures during the fusion of embryonic processes ^1^ ^,^ ^2^ and occurring between the 4th and 12th weeks of fetal development ^1^ ^,^ ^2^ ^,^ ^3^. They include cleft lip, resulting from failure to fuse the nasal processes with the maxillary process; cleft palate, resulting from failure to fuse, in the midline, the palatine processes; and cleft lip and palate, resulting from problems in the fusion of nasal prominences and maxillary processes ^1^ ^,^ ^3^. The term “orofacial clefts” covers all phenotypic variations of this condition, regardless of its classification, including cleft lip and palate, isolated cleft lip, or isolated cleft palate ^4^. They can occur as a syndromic component or as an isolated anomaly without another malformation present ^1^; these are referred to as nonsyndromic orofacial clefts ^5^.

The etiology of nonsyndromic orofacial clefts is multifactorial, involving an interaction between genetic factors and environmental exposures ^3^ ^,^ ^5^. Prevalence is influenced by geographic origin, racial and ethnic groups, environmental factors, and socioeconomic status ^3^. Worldwide, there is a variation between 3.8 and 5.7 million cases ^2^. The highest prevalence was observed in Asian and Amerindian populations, at approximately 2 per 1 thousand; intermediate prevalence was observed in European populations (1 per 1 thousand), while the lowest frequency was observed in African populations, at 0.4 per 1 thousand ^6^.

In Brazil, a prevalence of 0.51 per 1 thousand live births with nonsyndromic orofacial clefts is estimated, with the highest prevalence in the South (0.63 per 1 thousand), followed by the Southeast (0.42 per 1 thousand), the Central-West (0.40 per 1 thousand), the North (0.36 per 1 thousand) and, finally, the lowest in the Northeast (0.35 per 1 thousand) ^7^. However, there may be underreporting ^6^, as Acre, Amapá, Mato Grosso, Mato Grosso do Sul, Pará, Rio Grande do Norte, and Roraima reported a delay in initiating information collection on malformations ^7^.

The main treatments for individuals with nonsyndromic orofacial clefts include cheiloplasty, indicated for closing the cleft lip, which should be performed around 3 to 6 months old and with the patient weighing at least 5 kg; and palatoplasty, surgery performed to close the cleft palate, which should occur between 12 and 18 months old, with the patient weighing at least 10 kg ^8^ ^,^ ^9^. When surgeries are not performed at the ideal time, the physical, functional, aesthetic, and psychosocial needs of the individual are not fully met ^2^ ^,^ ^10^ ^,^ ^11^. In low- and middle-income countries, access to corrective surgery is limited due to economic constraints and social issues, including a lack of medical services, inadequate awareness, and cultural barriers ^2^.

Although different studies have evaluated patients with nonsyndromic orofacial clefts in Brazil, few have evaluated individuals from the Central-West Region ^12^. However, there may be difficulties in providing full multidisciplinary monitoring to individuals from this region, especially in the state of Mato Grosso, due to its vast territory ^10^. Thus, the present study aims to analyze the profile of individuals with nonsyndromic orofacial clefts from a reference service in Brazil. It is proposed as a study hypothesis that the creation of the center contributed to addressing an unmet demand for patients who did not have access to appropriate care, and thus, over the years, there was a change in the profile of new patients admitted to the service.

## Methods

Study design

This study adopted a retrospective observational design, analyzing data collected between January 2005 and December 2023.

Setting

The setting of this study is the Cleft Lip and Palate Rehabilitation Service at the General Hospital of Cuiabá, located in Cuiabá, Mato Grosso. It is a national reference in the treatment of patients with orofacial clefts, as established by Ordinance No. 562, since October 2008 ^13^, and is a pioneer in the state, with its service commencing in November 2004. It has a multidisciplinary team of plastic surgeons, oral and maxillofacial surgeons, speech therapists, pediatric dentists, orthodontists, nutritionists, physical therapists and psychologists, among other professionals who, together with the help of the nongovernmental organization Smile Train, work to improve the quality of life of approximately 1,460 patients with nonsyndromic orofacial clefts, registered until December 2023.

The data collection was performed between December 2023 and January 2024 by a single dentist, B. A. G., in the second year of a multidisciplinary residency in hospital care of patients with special needs, and considered data from January 2005 to December 2023. During this period, the data of patients treated at the service were evaluated, focusing on the information extracted from the medical records and evaluation forms used in the initial medical assessment. The recruitment process included patients diagnosed with nonsyndromic orofacial clefts who were registered in the hospital system until December 2023, and data were collected retrospectively.

Participants

The inclusion criteria were to be a patient registered in the hospital during the study period, and to have a nonsyndromic cleft lip, cleft lip and palate, or cleft palate. Those with incomplete records, patients with submucous cleft, rare facial clefts, and individuals not from Mato Grosso were excluded.

Variables 

The variables analyzed were: age group <3 or ≥3 years old, sex, type of orofacial cleft (isolated cleft lip, cleft lip and palate, and isolated cleft palate), period of admission to the service, city of origin when admitted to the service (Cuiabá and Várzea Grande, and other cities), area of residence, and history of surgeries before being admitted to the program (no history of previous surgeries and history of previous surgery). Cuiabá and Várzea Grande were grouped because they constitute a conurbation; thus, patients from these cities were considered to belong to the service site and were analyzed differently from patients from other cities in the state.

Regarding the age of patients when admitted to the service, previous studies served as references, suggesting that ideally, patients with orofacial clefts should have their treatment initiated before the age of 3 ^9^. Therefore, patients were grouped into two categories: those under 3 years old and those aged 3 years old or older when they were admitted to the service.

For analysis, the years of patient admission to the service were divided into periods: 2005-2007, 2008-2010, 2011-2013, 2014-2016, 2017-2019, and 2020-2023. The last period has two particularities: it encompassed the COVID-19 pandemic period, during which fewer patients were admitted for treatment, and, unlike the other periods grouped by three years, it was grouped by four years. This division respects the chronological order and facilitates comparison between years, allowing for the identification of patterns and trends in a clearer and more structured manner.

Data source and measurement

Data were collected between December 2023 and January 2024, from medical records and evaluation forms of patients treated at the General Hospital of Cuiabá. The variables of interest were measured considering clinical data (type of cleft, history of previous surgeries, and periods of admission to the service) and demographic data (age group, city of origin, area of residence). The patients were categorized into two age groups: those under 3 years old and those 3 years old or older. The variables were recorded directly in the patients’ medical records and analyzed retrospectively. 

Bias control

Forms were standardized, and the professionals who filled out the forms received training.

Study size

A population-based study was conducted, encompassing all 1,460 individuals with nonsyndromic orofacial clefts who received care at the Cleft Lip and Palate Rehabilitation Service at the General Hospital of Cuiabá between January 2005 and December 2023.

Statistical methods

To verify whether the creation of the center contributed to addressing an unmet demand for patients without access to appropriate care, a descriptive analysis of the collected variables was performed before and after the center’s creation. Contingency analyses were conducted to explore the relationships between age group and the variables of city of origin, area of residence, and history of previous surgeries, as well as the relationship between periods of admission to the service and the variables of age group and history of previous surgeries. To determine whether there were changes in the patient profile over the years, a multivariate binomial logistic regression analysis was performed. It evaluated the relationship between admission to the service for children under 3 years old or 3 years old or older, and the variables of sex, type of cleft, city of origin, and area of residence. The JAMOVI program (jamovi.org) was used to perform all statistical analyses, with the significance level defined as p-value<0.05.

## Result

A total of 1,460 records of individuals with nonsyndromic orofacial clefts who received care at the Cleft Lip and Palate Rehabilitation Service at the General Hospital of Cuiabá, between January 2005 and December 2023, were analyzed. Of these, 43 were excluded because they had incomplete data. Therefore, 1,417 records met the inclusion criteria and correspond to the population of the present study.

The distribution of individuals, considering the city of origin, area of residence, gender, type of cleft, and previous surgical history, was analyzed according to the period in which their treatment was initiated. As for the city of origin, 64.2% of the patients were from inland cities of Mato Grosso, while 35.8% were from Cuiabá and Várzea Grande; 90.1% of the patients lived in the urban area and 9.9% in the rural area; 55.7% were male and 44.3% female; 48.3% of the patients had cleft lip and palate; 25.9%, cleft lip; and 25.8%, cleft palate; 42.5% patients had undergone previous surgery, and 57.5% had not (Table 1).

It was analyzed whether the age group in which the patient is admitted to the service is related to the city of origin, area of residence, and history of previous surgeries. Most of the new patients came from inland cities (64.2%), regardless of age group. Among those from inland cities, a significantly higher proportion of individuals were admitted to the service at under 3 years old (56.8%), while in the cities of Cuiabá and Várzea Grande there was a higher proportion of new patients admitted to the service at 3 years old or older (56.5%) (p-value 0.001). In the analysis of the relationship between the age at which patients are admitted to the service and their area of residence, no significant difference was found between the groups. Regardless of the age group at admission to the service, there was a predominance of patients from the urban area (90.1%). Among patients under 3 years old, a high proportion had not undergone surgery prior to admission to the service (83.4%), whereas the opposite was true for patients admitted who were 3 years old or older (90.5%) (p-value 0.001) (Table 2).

**Table 1:** Distribution of patients according to treatment initiation periods, in absolute (n) and relative (%) frequencies, by demographic variables. Cuiabá, 2024 (n=1,417)

Variable	1st period	2nd period	3rd period	4th period	5th period	6th period	Total
	n (%)	n (%)	n (%)	n (%)	n (%)	n (%)	n (%)
City							
Cuiabá and	174	93	54	55	63	69	508
Várzea Grande	(12.3)	(6.5)	(3.8)	(3.9)	(4.4)	(4.9)	(35.8)
Inland of Mato Grosso	243	189	110	106	117	114	909
	(17.1)	(13.3)	(7.8)	(7.5)	(8.3)	(10.2)	(64.2)
Area of residence							
Urban	383	260	139	147	164	183	1.276
	-27,1	(18.3)	(9.8)	(10.4)	(11.6)	(12.9)	(90.1)
Rural	34	22	25	14	16	30	141
	-2,4	(1.5)	(1.8)	(1.0)	(1.1)	(2.1)	(9.9)
Sex							
Female	187	120	74	69	81	96	627
	-13,2	(8.5)	(5.2)	(4.9)	(5.7)	(6.8)	(44.3)
Male	230	162	90	92	99	117	790
	-16,3	(11.4)	(6.3)	(6.5)	(6.9)	(8.3)	(55.7)
Type of cleft							
Lip	98	67	56	46	45	56	368
	-6,9	(4.7)	(3.9)	(3.3)	(3.2)	(3.9)	(25.9)
Lip and Palate	211	153	72	78	70	99	683
	-14,9	(10.8)	(5.1)	(5.5)	(5.0)	(7.0)	(48.3)
Palate	108	62	36	37	65	58	366
	-7,6	(4.4)	(2.5)	(2.6)	(4.6)	(4.1)	(25.8)
History of surgeries							
No surgery	159	150	116	105	129	156	815
	-11,2	(10.6)	(8.2)	(7.4)	(9.1)	(11.0)	(57.5)
Surgery	258	132	48	56	51	57	602
	-18,2	(9.3)	(3.4)	(4.0)	(3.6)	(4.0)	(42.5)

**Table 2:** Distribution of patients by age at admission to the program, according to city of origin, area of residence, and prior history of surgeries, in absolute (n) and relative (%) frequencies. Cuiabá, 2024 (n=1,417)

Variable	Age group <3	Age group ≥3	Total	p-value
City				
Cuiabá and Várzea Grande	221 (43.5)	287 (56.5)	508 (100)	0.001
Inland of Mato Grosso	516 (56.8)	393 (43.2)	909 (100)	
Area of residence				
Rural area	83 (58.9)	58 (41.1)	141 (100)	0.086
Urban area	654 (51.2)	622 (48.8)	1,276 (100)	
History of surgeries				0.001
Yes	57 (9.5)	545 (90.5)	602 (100)	
No	680 (83.4)	135 (16.6)	815 (100)	
Total	737 (52.0)	680 (48.0)	1,417 (100)	

The study observed that, in the first period, there was a higher admission rate of patients aged 3 years old or older and with a history of previous surgery; in the second period, there was a balance; and in the other periods, the admission of patients who had no previous surgeries and under 3 years old prevailed. Therefore, it is observed that there was a change in the profile of patients admitted to the service in the evaluated period (p-value 0.001) (Table 3).

Multivariate binomial logistic regression analysis showed that female patients are more likely to be admitted to the service at 3 years old or older than male patients (p-value 0.001). Regarding the type of cleft, no difference was found between patients with cleft lip and cleft lip and palate. On the other hand, patients with cleft palate are more likely to be admitted to the service at 3 years old or older than those with cleft lip and palate (p-value 0.013). Residents of Cuiabá and Várzea Grande are more likely to be admitted to the service at 3 years old or older than those from inland cities in Mato Grosso (p-value < 0.001). There was no significant difference between the age of admission to the service between residents of rural and urban areas (p-value 0.471) (Table 4).

**Table 3:** Distribution of patients by period of admission to the service, according to prior history of surgery and patient age group, in absolute (n) and relative (%) frequencies. Cuiabá, 2024 (n=1,417)

Variable	1^st^ period	2^nd^ period	3^rd^ period	4^th^ period	5^th^ period	6^th^ period	Total	p-value
Previous history of surgery								
Yes	258 (61.9)	132 (46.8)	48 (29.3)	56 (34.8)	51 (28.3)	57 (26.8)	602 (42.5)	<0.001
No	159 (38.1)	150 (53.2)	116 (70.7)	105 (65.2)	129 (71.7)	156 (73.2)	815 (57.5)	
Total	417 (100)	282 (100)	164 (100)	161 (100)	180 (100)	213 (100)	1,417 (100)	
Age group (years)								
<3	131 (31.4)	144 (51.0)	98 (59.8)	98 (60.9)	112 (62.2)	154 (72.3)	737 (52.0)	
≥3	286 (68.6)	138 (49.0)	66 (40.2)	63 (39.1)	68 (37.8)	59 (27.7)	680 (48.0)	
**Total**	417 (100)	282 (100)	164 (100)	161 (100)	180 (100)	213 (100)	1,417 (100)	<0.001

**Table 4:** Binomial logistic regression considering the variables: age group (<3 or ≥3 years old), sex, type of cleft, city of origin, and area of residence, with 95% confidence intervals (95% CI). Cuiabá, 2024 (n=1,417).

Variable	Estimate	Standard Error	z-score	p-value	Odds ratio (95%CI)
Sex					
Female (age group ≥3 years old) versus male (age group ≥3 years old)	0.36	0.11	3.26	0.001	1.43 (1.15; 1.77)
Type of cleft					
Lip (age group ≥3 years old) vs*.* lip and palate (age group ≥3 years old)	-0.21	0.13	-1.60	0.110	0.81 (0.62; 1.04)
Palate (age group ≥3 years old) vs. lip and palate (age group ≥3 years old)	-0.33	0.13	-2.48	0.013	0.71 (0.55; 0.93)
City					
Cuiabá and Várzea Grande (age group ≥3 years old) vs. Inland of Mato Grosso (age group ≥3 years old)	0.51	0.12	4.46	<0.001	1.67 (1.33; 2.09)
Area of residence					
Urban (age group ≥3 years old) vs. rural (age group ≥3 years old)	0.13	0.19	0.72	0.471	1.14 (0.79; 1.64)

## Discussion

The profile of new patients admitted to the analyzed service is predominantly male, with cleft lip and palate, from urban areas of cities in the inland regions of the state, and with no history of prior surgical procedures. However, this profile underwent significant changes in the period studied. In the first years of the service’s operation, individuals who were 3 years old or older with a history of previous surgeries predominated. In the later periods, new patients were predominantly younger than 3 years old and had no history of surgeries, confirming the research hypothesis.

This research has inherent limitations to the design of retrospective observational studies ^14^. Data collection was conducted using medical records and evaluation forms completed over time by various professionals to assist, rather than through formal research. However, the standardization of forms and the training of professionals minimize distortions in the records. Another limitation is the convenience sampling, which can be influenced by external factors, such as access to the service, cultural habits, and environmental factors ^15^ specific to the patients’ region of origin. In addition, the inclusion of data from a single hospital, although a reference in the state, may be a limiting factor. However, similar studies conducted in single centers have proved to be important sources of information ^9^ ^,^ ^16^. 

The incidence of orofacial clefts varies among different population groups ^5^, with cleft lip and palate, as well as isolated cleft lip, being more frequent in men, and isolated cleft palate being more frequent in women ^17^. Although this is a convenience sample, the higher prevalence of male patients with nonsyndromic cleft lip and palate found in this study aligns with other surveys conducted in Brazil ^5^ ^,^ ^6^ ^,^ ^7^ ^,^ ^17^ ^,^ ^18^ and in other countries ^16^. Brazilian surveys indicated a prevalence of cleft lip and palate in males 2.57 to 2.78 times higher than in females ^17^ ^,^ ^18^. A hypothesis raised for this higher prevalence in males would be due to specific susceptibility factors in the X or Y chromosomes, which could influence the occurrence of cleft lip and palate in males - such as the compensatory second copy of the X chromosome, the lack of a Y chromosome ^19^, or the inactivation of the X chromosome in women ^20^. However, this theory lacks confirmation ^19^ ^,^ ^20^. A study of the Chilean population found evidence of an association between nonsyndromic cleft lip and palate and male sex, attributed to a variation in the MSX1 gene ^21^. This study identified that male patients are admitted to the service at an earlier age than female patients, possibly due to the external location of the cleft lips and cleft lips and palates, more common in males ^5^ ^,^ ^6^ ^,^ ^7^ ^,^ ^17^ ^,^ ^18^, which contributes to its early detection ^22^, allowing the immediate start of preparation for surgery ^11^. In contrast, cleft palates, which are more prevalent in females ^(7)^, are more difficult to diagnose due to their intraoral location ^22^.

In 2024, the estimated population of Mato Grosso was 3,836,399 inhabitants, with 682,932 in Cuiabá and 314,627 in Várzea Grande ^23^, reflecting the population distribution within the state, which may help explain the origin of the registered patients. In addition, 82% of Mato Grosso’s inhabitants live in urban areas ^24^, which may explain the predominance of individuals from urban areas, as observed in other studies conducted in Brazil ^25^. The higher prevalence of patients with orofacial clefts in urban areas may also be related to the distance between the rural population and health services, which may hinder their access to treatment ^12^ ^,^ ^25^.

Most patients from the inland region of Mato Grosso were admitted to the rehabilitation service before the age of 3, while those from Cuiabá and Várzea Grande were admitted at 3 years old or older. When an individual is born or diagnosed with an untreatable condition in their city, health facilities refer cases to the service closest to their residence via the National Health Regulation System ^26^. Therefore, individuals with nonsyndromic orofacial clefts in the state of Mato Grosso are preferably treated in Cuiabá ^12^. This may explain why patients from the inland region of the state are admitted at the appropriate age for treatment. However, patients from Cuiabá and Várzea Grande who were already being monitored in other states may have chosen to transfer to the Cleft Lip and Palate Rehabilitation Service at the General Hospital of Cuiabá after its implementation, explaining the inclusion of individuals who are older and have undergone previous surgeries.

Whether the patient resides in an urban or rural area did not affect the age at which they were admitted to the service. Although the scarcity of rural infrastructure may delay diagnosis and treatment ^27^, this was not identified in this study. Three possible reasons for this would be the fast identification of facial malformations, unlike malformations in internal organs ^28^; the formal reference in the state health system for referral of patients with orofacial clefts ^26^; and the fact that the General Hospital of Cuiabá is a state reference for high-risk childbirth ^13^, since there are cases of intrauterine diagnosis of orofacial clefts through ultrasonography ^22^. As a result, since 2008, the General Hospital has begun to systematically receive patients born with the deformity in the state ^13^. 

A higher admission rate of individuals 3 years old or older with a history of surgeries was observed in the earlier periods, whereas in the later periods, admissions were predominantly patients under 3 years old without previous surgeries. This reflects the situation prior to the creation of the service, when patients needed to seek care in services distributed across Brazil, due to the absence of a specialized service in Mato Grosso. During this period, distance and lack of monitoring made treatment difficult, and many patients received only surgical treatment and were left unassisted ^12^. In 1999, Operation Smile carried out “Operação Sorriso Brasil” in the Central-West, starting in Cuiabá ^29^, leaving several patients without multidisciplinary post-surgery monitoring. This also helps explain why the first patients registered in the rehabilitation service had already undergone surgery, whereas, as of the second period, patients were mostly admitted to begin treatment without prior surgery.

The change in the profile of new patients to the Cleft Lip and Palate Rehabilitation Service at the General Hospital of Cuiabá during the studied period can be attributed to the implementation of the service itself, which facilitated access to treatment and managed the accumulated local demand. As a result, patients began to seek care in the first years of life. The anticipation of the start of treatment was also observed in Belém, state of Pará, where the number of surgeries performed at the ideal time increased by 12% between the first and last four years of service ^8^.

However, a study evaluating 28 Brazilian hospitals accredited for the treatment of orofacial clefts reveals regional disparities, especially in the North, Northeast, and Central-West regions, which still face limited service availability ^30^. In the case of Mato Grosso, the state’s large territory and the distance of its cities from the capital may hinder full multiprofessional monitoring ^10^, as treatment requires frequent consultations over time for the complete restoration of these individuals ^11^. This difficulty in access can compromise the effectiveness of treatment and the prognosis of patients. Therefore, it is recommended that further studies be conducted in order to evaluate the need to implement other decentralized services to meet this possible demand; studies that include different centers, in order to analyze whether the profile of the patients is in accordance with what was found; and whether the individuals of the studied center with previous treatment received the intervention at the proper age, the places where the treatments were performed and the most common surgical procedures. It is also suggested to include details on laterality (unilateral, bilateral) and cleft subtypes (complete, incomplete) in future studies. 

The analysis of patient profiles admitted to the Cleft Lip and Palate Rehabilitation Service at the General Hospital of Cuiabá reveals a predominance of male individuals with cleft lip and palate from the urban area of the inland of Mato Grosso, with no history of previous surgical procedures. The profile has evolved, transitioning from a higher admission rate of older patients with a history of prior surgery to a higher admission rate of younger patients without prior surgery.
