# Supplementary material for: Analysis of the profile of patients admitted to an orofacial cleft rehabilitation service: An observational study, Cuiabá, 2005-2023
Source: Epidemiol Serv Saude. 2025 Oct 24;34:e20240926. doi: 10.1590/S2237-96222025v34e20240926.en (PMC12551946; doi:10.1590/S2237-96222025v34e20240926.en)
Supplement: Translation

# Translation: Análise do perfil de pacientes ingressantes em serviço de reabilitação de fissuras orofaciais: estudo observacional, Cuiabá, 2005-2023

**DOI:** 10.1590/S2237-96222025v34e20240926.pt

**Authors:** Brunna Almeida Guerreiro, Lorraynne dos Santos Lara, Ivan Onone Gialain, Carolina Silvano Vilarinho da Silva, Luiz Evaristo Ricci Volpato

**Affiliations:** 1 Universidade de Cuiabá, Programa de Pós-graduação em Ciências Odontológicas Integradas, Cuiabá, MT, Brasil; 2 Hospital Geral de Cuiabá, Departamento de Odontologia, Cuiabá, MT, Brasil; 3 Hospital de Câncer de Mato Grosso, Departamento de Odontologia, Cuiabá, MT, Brasil

**Keywords:** Epidemiologia, Equipe de Assistência ao Paciente, Fenda Labial, Fissura Palatina, Odontologia em Saúde Pública

## Abstract

**Objetivo:**

Analisar o perfil de indivíduos com fissuras orofaciais não sindrômicas de um serviço de referência no Brasil.

**Métodos:**

Este estudo observacional retrospectivo com pacientes ingressantes no Serviço de Reabilitação de Fissuras Labiopalatinas do Hospital Geral de Cuiabá, Mato Grosso, entre 2005 e 2023, considerou faixa etária, sexo, tipo de fissura, município de residência, zona de procedência, histórico cirúrgico e ano de ingresso. Os dados foram obtidos a partir dos registros clínicos. Realizou-se análise descritiva das variáveis, com frequências absolutas e relativas, e regressão logística binomial, considerando faixa etária <3 anos ou ≥3 anos de idade e sexo, tipo de fissura, município de origem e zona de procedência, com intervalos de confiança de 95%.

**Resultados:**

Dos 1.417 pacientes analisados, 55,7% era do sexo masculino, 48,2% com fissura labiopalatina, 90,0% provenientes da zona urbana, 64,1% de municípios do interior mato-grossense e 57,5% sem histórico de cirurgia prévia. O perfil dos pacientes sofreu significativas mudanças no período estudado. Nos primeiros anos, prevaleceram indivíduos com ≥3 anos (68,5%), com cirurgias prévias (61,8%), enquanto nos últimos períodos prevaleceram os indivíduos menores de 3 anos (72,3%), sem cirurgias (73,2%).

**Conclusão:**

O perfil dos pacientes revela predomínio de indivíduos do sexo masculino, com fissura labiopalatina, provenientes de municípios do interior de Mato Grosso, residentes na zona urbana e sem histórico cirúrgico. Esse perfil mudou com o tempo, passando de um ingresso maior de pacientes mais velhos e com cirurgia prévia, nos primeiros anos, para pacientes mais jovens e sem cirurgia prévia nos últimos anos analisado.

**Aspectos éticos**

Esta pesquisa respeitou os princípios éticos, obtendo os seguintes dados de aprovação:

Comitê de ética em pesquisa: Universidade de Cuiabá

Número do parecer: 3.610.573

Data de aprovação: 30/9/2019

Certificado de apresentação de apreciação ética: 21617419.9.0000.5165

Registro do consentimento livre e esclarecido: Obtido de todos os participantes antes da coleta.

## Introdução

As fissuras orofaciais são as anomalias congênitas mais comuns que acometem o ser humano, originadas de falhas durante a fusão dos processos embrionários ^1^ ^,^ ^2^, e ocorrem entre a 4a e a 12a semanas de desenvolvimento intrauterino ^1^ ^,^ ^2^ ^,^ ^3^. Elas compreendem a fissura labial, resultante da falha na fusão dos processos nasais com o processo maxilar; a fissura palatina, resultante da falha de fusão, na linha mediana, dos processos palatinos; e as fissuras labiopalatinas, decorrentes de problemas na fu são das proeminências nasais e processos maxilares ^1^ ^,^ ^3^. O termo “fissuras orofaciais” abrange todas as variações fenotípicas dessa condição, independentemente de sua classificação, incluindo fissuras labiopalatinas, labiais e palatinas isoladas ^4^. Elas podem ocorrer como um componente sindrômico ou como uma anomalia isolada, sem outra malformação presente ^1^; esta, nomeamos de fissura orofacial não sindrômica ^5^. 

A etiologia das fissuras orofaciais não sindrômicas é multifatorial, envolvendo uma interação entre fatores genéticos e exposições ambientais ^3^ ^,^ ^5^. A prevalência é influenciada pela origem geográfica, grupos raciais e étnicos, fatores ambientais e status socioeconômico ^3^. Mundialmente, existe uma variação entre 3,8 e 5,7 milhões de casos ^2^. As maiores prevalências foram observadas nas populações asiáticas e ameríndias, em torno de 2/1 mil; com prevalência intermediária, vêm as populações euro peias (1/1 mil), enquanto a menor frequência é observada nas populações africanas, com 0,4/1 mil ^6^. 

No Brasil, estima-se uma prevalência de 0,51/1 mil nascidos vivos com fissuras orofaciais não sindrômicas, sendo a maior prevalência na região Sul (0,63/1 mil), seguida pelo Sudeste (0,42/1 mil), o Centro-Oeste (0,40/1 mil), o Norte (0,36/1 mil) e, por fim, a menor no Nordeste (0,35/1 mil) ^7^. No entanto, pode haver sub notificação ^6^, já que Acre, Amapá, Mato Grosso, Mato Grosso do Sul, Pará, Rio Grande do Norte e Roraima reconhecidamente tiveram atraso no início da coleta de informações sobre malformações ^7^. 

Os principais tratamentos para indivíduos com fissu ras orofaciais não sindrômicas incluem a queiloplastia, indicada para o fechamento da fissura do lábio, que deve ser realizada por volta dos 3 a 6 meses de vida e com o paciente pesando no mínimo 5 kg; e a palatoplastia, cirurgia realizada para fechamento da fissura do palato, que deve ocorrer entre 12 e 18 meses de vida, com o paciente pesando no mínimo 10 kg ^8^ ^,^ ^9^. Quando as cirurgias não são realizas no momento considerado ideal, as necessidades físicas, funcionais, estéticas e psicossociais do indivíduo não são plenamente aten didas ^2^ ^,^ ^10^ ^,^ ^11^. Nos países de baixa e média renda, há limitação ao acesso às cirurgias corretivas, por razões econômicas e problemas sociais, como deficiência dos serviços médicos, ignorância e influência cultural ^2^. 

Embora diferentes estudos tenham avaliado pacientes com fissuras orofaciais não sindrômicas no Brasil, poucos avaliaram indivíduos da região Centro-Oeste ^12^. No en tanto, pode haver dificuldade no pleno acompanhamento multiprofissional dos indivíduos dessa região, especial mente no estado de Mato Grosso, devido ao seu vasto território ^10^. Assim, o presente estudo tem como objetivo analisar o perfil de indivíduos com fissuras orofaciais não sindrômicas de um serviço de referência no Brasil. Apresenta-se como hipótese de estudo que a criação do centro contribuiu para suprir uma demanda reprimida de pacientes que não tinham acesso ao atendimento adequado, e assim, com o passar dos anos, houve uma mudança no perfil dos pacientes ingressantes no serviço. 

## Métodos

Desenho do estudo 

Este estudo adotou um desenho observacional re trospectivo, analisando dados coletados entre janeiro de 2005 e dezembro de 2023.

Contexto 

O cenário deste estudo é o conduzido no Serviço de Reabilitação de Fissuras Labiopalatinas do Hospital Geral de Cuiabá, localizado em Cuiabá, Mato Grosso. Ele é referência nacional no tratamento dos pacientes com fissuras orofaciais pela portaria n° 562, desde outubro de 2008 ^13^, sendo pioneiro no estado, com início do serviço em novembro de 2004. Conta com uma equi pe multiprofissional de cirurgiões plásticos, cirurgiões bucomaxilofaciais, fonoaudiólogos, odontopediatras, ortodontistas, nutricionistas, fisioterapeutas e psicólo gos, entre outros profissionais que, juntamente com o auxílio da organização não governamental Smile Train, trabalham para melhorar a qualidade de vida de apro ximadamente 1.460 pacientes com fissuras orofaciais não sindrômicas, cadastrados até dezembro de 2023. 

A coleta de dados foi realizada entre dezembro de 2023 e janeiro de 2024, por uma única cirurgiã-dentista, B.A.G., no segundo ano de residência multiprofissional em saúde hospitalar de pacientes com necessidades especiais, e considerou dados de janeiro de 2005 a de zembro de 2023. Durante este período, foram avaliados os dados de pacientes atendidos no serviço, com foco nas informações extraídas dos prontuários médicos e fichas de avaliação das consultas de triagem. O proces so de recrutamento incluiu pacientes diagnosticados com fissuras orofaciais não sindrômicas, cadastrados no sistema do hospital até dezembro de 2023, e os dados foram coletados de forma retrospectiva. 

Participantes 

Foram considerados como critérios de inclusão ser paciente cadastrado no hospital no período de estudo, e possuir fissura labial, fissura labiopalatina ou fissura palatina manifestadas de forma não sindrômica. Foram excluídos aqueles que estavam com fichas incompletas, pacientes com fissura submucosa, fissuras raras de face e indivíduos não oriundos de Mato Grosso. 

Variáveis 

As variáveis analisadas foram: faixa etária <3 ou ≥3, sexo, tipo de fissura orofacial (fissura labial isolada, fissura labiopalatina e fissura palatina isolada), período de ingresso no serviço, município de origem ao dar entrada no serviço (Cuiabá e Várzea Grande e outros municípios), zona de procedência, e histórico cirúrgico prévio ao ingresso no programa (sem histórico cirúrgico prévio e com histórico de cirurgia prévia). Cuiabá e Várzea Grande foram agrupadas por constituírem uma conurbação urbana; dessa forma, os pacientes desses municípios foram considerados como pertencentes ao local do serviço e analisados de forma distinta dos pacientes de outros municípios do estado. 

Com relação à idade dos pacientes ao ingressar no serviço, utilizaram-se como referências estudos ante riores que consideraram que, idealmente, os pacientes com fissuras orofaciais deveriam ter seu tratamento iniciado antes dos 3 anos de idade ^9^. Assim, os pa cientes foram agrupados em menores de 3 anos e com 3 anos ou mais, ao ingressar no serviço. 

Para análise, os anos de ingresso dos pacientes no serviço foram divididos em períodos, de 2005 a 2007, de 2008 a 2010, de 2011 a 2013, de 2014 a 2016, de 2017 a 2019 e de 2020 a 2023. O último período conta com duas particularidades: incluiu o período da pan demia da covid-19, com menor ingresso de pacientes para tratamento e, diferentemente dos demais períodos que foram de triênios, foi composto por quatro anos. Essa divisão respeita a ordem cronológica e facilita a comparação entre os anos, permitindo identificar padrões e tendências de forma mais clara e estruturada. 

Fontes de dados e mensuração 

Os dados foram coletados entre dezembro de 2023 e janeiro de 2024, a partir de prontuários médicos e fichas de avaliação de pacientes atendidos no Hospital Geral de Cuiabá. As variáveis de interesse foram men suradas considerando-se os dados clínicos (tipo de fissura, histórico de cirurgias prévias e períodos de ingresso no serviço) e demográficos (faixa etária, município de origem, zona de procedência). A idade dos pacientes foi categorizada em duas faixas: menor que 3 anos e 3 anos ou mais. As variáveis foram re gistradas diretamente nos prontuários dos pacientes e analisadas retrospectivamente.

Controle de viés 

Houve padronização dos formulários e o treinamento dos profissionais que preencheram os formulários. 

T amanho do estudo 

Foi conduzido um estudo censitário, abrangendo todos os 1.460 indivíduos com fissuras orofaciais não sindrômicas que receberam atendimento no Serviço de Reabilitação de Fissuras Labiopalatinas do Hospital Geral de Cuiabá, entre janeiro de 2005 e dezembro de 2023. 

Métodos estatísticos 

Para verificar se a criação do centro contribuiu para suprir uma demanda reprimida de pacientes sem aces so ao atendimento adequado, f oi realizada a análise descritiva das variáveis coletadas antes e depois da criação do centro. Também foram conduzidas análises contingenciais que exploraram a relação entre faixa etária e as variáveis município de origem, zona de procedência e histórico de cirurgias prévias, e a relação entre os períodos de ingresso no serviço e as variáveis faixa etária e histórico de cirurgias prévias. Para iden tificar se houve mudanças no perfil dos pacientes ao longo dos anos, foi realizada análise multivariada de regressão logística binomial. Ela avaliou a relação entre o ingresso no serviço com idade inferior a 3 anos ou com 3 anos de idade ou mais, e as variáveis sexo, tipo de fissura, município de origem e zona de procedência. O programa JAMOVI (jamovi.org) foi utilizado para realizar todas as análises estatísticas, com o nível de significância definido como p-valor<0,05. 

## Resultados

Foram analisadas 1.460 fichas de indivíduos com fissuras orofaciais não sindrômicas que receberam atendimento no Serviço de Reabilitação de Fissuras Labiopalatinas do Hospital Geral de Cuiabá, entre ja neiro de 2005 e dezembro de 2023. Dessas, 43 foram excluídas porque estavam com dados incompletos; assim, 1.417 fichas atenderam aos critérios de inclusão e correspondem à população do presente estudo. 

A distribuição dos indivíduos, considerando-se o município de origem, zona de procedência, sexo, tipo de fissura e histórico cirúrgico prévio, foi analisada de acordo com o período em que o seu tratamento foi iniciado. Quanto ao município de origem, 64,2% dos pacientes eram do interior de Mato Grosso, enquanto 35,8% eram de Cuiabá e Várzea Grande; 90,1% dos pacientes residiam na zona urbana e 9,9% na zona rural; 55,7% eram do sexo masculino e 44,3% do sexo feminino; 48,3% dos pacientes tinham fissura labiopa latina; 25,9%, fissura labial; e 25,8%, fissura palatina; 42,5% pacientes haviam realizado cirurgia prévia, e 57,5%, não (Tabela 1). 

A nalisou-se se a faixa etária em que o paciente ingressa no serviço está relacionada ao município de origem, zona de procedência, e histórico de cirurgias prévias. A maioria dos ingressantes veio do interior (64,2%), independentemente da faixa etária. Entre os oriundos do interior, uma proporção significativamente maior de indivíduos ingressou no serviço com idade inferior a 3 anos (56,8%), enquanto nos municípios de Cuiabá e Várzea Grande houve maior proporção de ingressantes no serviço com 3 anos de idade ou mais (56,5%) (p-valor 0,001). Na análise da relação entre a idade em que o paciente inicia no serviço e a zona de procedência, não foi encontrada diferença significativa entre os grupos. Independentemente da faixa etária de ingresso no serviço, houve predomínio de pacientes da zona urbana (90,1%). Entre os pacientes com idade inferior a 3 anos, houve um elevado número deles que não haviam sido submetidos a cirurgias previamente ao ingresso no serviço (83,4%), o contrário ocorrendo entre os pacientes ingressantes com 3 anos de idade ou mais (90,5%) (p-valor 0,001) (Tabela 2).

**Tabela 1:** Distribuição dos pacientes de acordo com os períodos de início do tratamento, em frequências absoluta (n) e relativa (%), segundo variáveis demográficas. Cuiabá, 2024 (n=1.417)

Variável	1º período	2º período	3º período	4º período	5º período	6º período	Total
	n (%)	n (%)	n (%)	n (%)	n (%)	n (%)	n (%)
Município							
Cuiabá e	174	93	54	55	63	69	508
Várzea Grande	-12,3	-6,5	-3,8	-3,9	-4,4	-4,9	-35,8
Interior de Mato Grosso	243	189	110	106	117	114	909
	-17,1	-13,3	-7,8	-7,5	-8,3	-10,2	-64,2
Zona de procedência							
Urbana	383	260	139	147	164	183	1.276
	-27,1	-18,3	-9,8	-10,4	-11,6	-12,9	-90,1
Rural	34	22	25	14	16	30	141
	-2,4	-1,5	-1,8	-1	-1,1	-2,1	-9,9
Sexo							
Feminino	187	120	74	69	81	96	627
	-13,2	-8,5	-5,2	-4,9	-5,7	-6,8	-44,3
Masculino	230	162	90	92	99	117	790
	-16,3	-11,4	-6,3	-6,5	-6,9	-8,3	-55,7
Tipos de fissura							
Labial	98	67	56	46	45	56	368
	-6,9	-4,7	-3,9	-3,3	-3,2	-3,9	-25,9
Labiopalatina	211	153	72	78	70	99	683
	-14,9	-10,8	-5,1	-5,5	-5	-7	-48,3
Palatina	108	62	36	37	65	58	366
	-7,6	-4,4	-2,5	-2,6	-4,6	-4,1	-25,8
Histórico cirúrgico							
Não operado	159	150	116	105	129	156	815
	-11,2	-10,6	-8,2	-7,4	-9,1	-11	-57,5
Operado	258	132	48	56	51	57	602
	-18,2	-9,3	-3,4	-4	-3,6	-4	-42,5

**Tabela 2:** Relação dos pacientes de acordo com a faixa etária de ingresso no programa, segundo o município de origem, a zona de procedência e o histórico cirúrgico prévio, em frequências absoluta (n) e relativa (%). Cuiabá, 2024 (n=1.417)

Variável	Faixa etária <3	Faixa etária ≥3	Total	p-valor
Município				
Cuiabá e Várzea Grande	221 (43,5)	287 (56,5)	508 (100)	0,001
Interior de Mato Grosso	516 (56,8)	393 (43,2)	909 (100)	
Zona de Procedência				
Zona rural	83 (58,9)	58 (41,1)	141 (100)	0,086
Zona urbana	654 (51,2)	622 (48,8)	1.276 (100)	
Histórico cirúrgico				
Sim	57 (9,5)	545 (90,5)	602 (100)	0,001
Não	680 (83,4)	135 (16,6)	815 (100)	
Total	737 (52,0)	680 (48,0)	1.417 (100)	

Observou-se que, no primeiro período, houve maior ingresso de pacientes com 3 anos de idade ou mais e de pacientes com histórico de cirurgia prévia; no segundo período, houve um equilíbrio; e nos demais períodos, prevaleceu o ingresso de pacientes sem cirurgias prévias e com idade menor que 3 anos. Observa-se, assim, que ocorreu uma mudança no perfil dos pacientes ingressantes no serviço no período avaliado (p-valor 0,001) (Tabela 3). 

A análise multivariada de regressão logística binomial demonstrou que as pacientes do sexo feminino têm mais chances de ingressar no serviço com 3 anos de idade ou mais do que os pacientes do sexo masculino (p-valor 0,001). Quanto ao tipo de fissura, não foi encontrada diferença entre os pacientes com fissura labial e fissura labiopalatina. Já os pacientes com fissura palatina têm mais chances de ingressar no serviço com 3 anos de idade ou mais do que aqueles com fissura labiopalatina (p-valor 0,013). Moradores de Cuiabá e Várzea Grande têm mais chances de ingressar no serviço com idade igual ou superior a 3 anos do que aqueles do interior de Mato Grosso (p-valor<0,001). Não houve diferença significativa entre a idade de ingresso no serviço entre residentes da zona rural e da zona urbana (p-valor 0,471) (Tabela 4). 

**Tabela 3:** Relação dos pacientes de acordo com o período de ingresso no serviço, segundo o histórico cirúrgico prévio e a faixa etária do paciente, em frequências absoluta (n) e relativa (%). Cuiabá, 2024 (n=1.417)

**Variável**	**1º período**	**2º período**	**3º período**	**4º período**	**5º período**	**6º período**	**Total**	**p-valor**
**Histórico cirúrgico prévio**								
Sim	258 (61,9)	132 (46,8)	48 (29,3)	56 (34,8)	51 (28,3)	57 (26,8)	602 (42,5)	<0,001
Não	159 (38,1)	150 (53,2)	116 (70,7)	105 (65,2)	129 (71,7)	156 (73,2)	815 (57,5)	
Total	417 (100)	282 (100)	164 (100)	161 (100)	180 (100)	213 (100)	1.417 (100)	
**Faixa etária (anos)**								
<3	131 (31,4)	144 (51,0)	98 (59,8)	98 (60,9)	112 (62,2)	154 (72,3)	737 (52,0)	
≥3	286 (68,6)	138 (49,0)	66 (40,2)	63 (39,1)	68 (37,8)	59 (27,7)	680 (48,0)	
**Total**	417 (100)	282 (100)	164 (100)	161 (100)	180 (100)	213 (100)	1.417 (100)	<0,001

**Tabela 4:** Regressão logística binomial, considerando-se pelas variáveis faixa etária menor que 3 anos ou com 3 anos de idade ou mais, sexo, tipo de fissura, município de origem e zona de procedência, com intervalos de confiança de 95% (IC95%). Cuiabá, 2024 (n=1.417)

**Variável**	**Estimativa**	**Erro padrão**	**Valor Z**	**p-valor**	**Odds ratio (IC95%)**
**Sexo**					
Feminino (faixa etária ≥3 anos) versus masculino (faixa etária ≥3 anos)	0,36	0,11	3,26	0,001	1,43 (1,15; 1,77)
**Tipo de fissura**					
Labial (faixa etária ≥3 anos) vs. labiopalatina (faixa etária ≥3 anos)	-0,21	0,13	-1,6	0,11	0,81 (0,62; 1,04)
Palatina (faixa etária ≥3 anos) vs. labiopalatina (faixa etária ≥3 anos)	-0,33	0,13	-2.48	0,013	0,71 (0,55; 0,93)
**Município**					
Cuiabá e Várzea Grande (faixa etária ≥3 anos) vs. Interior de Mato Grosso (faixa etária ≥3 anos)	0,51	0,12	4,46	<0,001	1,67 (1,33; 2,09)
**Procedência**					
Urbana (faixa etária ≥3 anos) vs. rural (faixa etária ≥3 anos)	0,13	0,19	0,72	0,471	1,14 (0,79; 1,64)

## Discussão

O perfil dos pacientes ingressantes no serviço analisado é majoritariamente masculino, com fissura la biopalatina, provenientes da zona urbana de municípios do interior do estado e sem histórico de procedimento cirúrgico prévio. Entretanto, esse perfil sofreu signifi cativas mudanças no período estudado. Nos primeiros anos de funcionamento do serviço, prevaleciam os indivíduos com 3 anos de idade ou mais e com histórico de cirurgias prévias, enquanto nos últimos períodos os ingressantes eram majoritariamente menores de 3 anos e sem histórico de cirurgias, confirmando a hipótese da pesquisa. 

Esta pesquisa possui limitações inerentes ao dese nho dos estudos observacionais retrospectivos ^14^. A coleta de dados foi realizada a partir de prontuários e fichas de avaliação preenchidos, ao longo do tempo, por diferentes profissionais, com intuito assistencial, não de pesquisa. No entanto, a padronização dos for mulários e o treinamento dos profissionais minimizam distorções nos registros. Outra limitação é a amostra por conveniência, que pode ser influenciada por fatores externos, como o acesso ao serviço, hábitos culturais e fatores ambientais ^15^ específicos da região de origem dos pacientes. Além disso, a inclusão de dados de um único hospital, embora referência no estado, pode ser um fator de restrição. Entretanto, estudos semelhantes realizados em centros únicos têm se mostrado impor tantes fontes de informação ^9^ ^,^ ^16^. 

A incidência de fissuras orofaciais varia entre diferentes grupos populacionais ^5^, com fissuras la biopalatinas e labiais isoladas sendo mais frequentes em homens, e fissura palatina isolada, em mulheres ^17^. Apesar de se tratar de uma amostra por conveniência, a maior prevalência de pacientes do sexo masculino e com fissura labiopalatina não sindrômica, encon trada neste estudo, está em consonância com outros levantamentos realizados no Brasil ^5^ ^,^ ^6^ ^,^ ^7^ ^,^ ^17^ ^,^ ^18^ e em outros países ^16^. Levantamentos brasileiros indicaram prevalência de fissura labiopalatina no sexo masculino de 2,57 a 2,78 vezes superior ao feminino ^17^ ^,^ ^18^. Uma hipótese levantada para essa maior prevalência no sexo masculino seria devido a fatores de suscetibilidade específicos nos cromossomos X ou Y, que poderiam influenciar a ocorrência de fissura labiopalatina no sexo masculino - como a segunda cópia compensatória do cromossomo X, a falta de um cromossomo Y ^19^, ou ainda, a inativação do cromossomo X em mulheres ^20^. Todavia, essa teoria carece de confirmação ^19^ ^,^ ^20^. Um estudo com a população chilena encontrou evidências de associação entre a fissura labiopalatina não sindrômica e o sexo masculino devido a uma va riação no gene MSX1 ^21^. Este estudo identificou que os pacientes masculinos ingressam no serviço em idade mais precoce do que as pacientes femininas, possivelmente devido à localização externa das fissuras labiais e labiopalatinas, mais comuns no sexo masculino ^5^ ^,^ ^6^ ^,^ ^7^ ^,^ ^17^ ^,^ ^18^, o que facilita sua identificação precoce ^22^, permitindo o início imediato do preparo para a cirurgia ^11^. Em contraste, as fissuras palatinas, mais prevalentes no sexo feminino ^7^, são mais difíceis de diagnosticar, devido à sua localização intraoral ^22^. 

Em 2024, a estimativa da população de Mato Grosso era de 3.836.399 habitantes, sendo 682.932 em Cuiabá e 314.627 em Várzea Grande ^23^, refletindo a dispersão populacional no interior do estado, o que pode justificar a origem dos pacientes cadastrados. Além disso, 82% dos habitantes de Mato Grosso residem em áreas ur banas ^24^, o que pode esclarecer a predominância de indivíduos oriundos da zona urbana, como em outros estudos realizados no Brasil ^25^. A maior prevalência de pacientes com fissuras orofaciais nas áreas urbanas também pode estar relacionada à distância entre a população rural e os serviços de saúde, possivelmente dificultando seu acesso ao tratamento ^12^ ^,^ ^25^. 

A maioria dos pacientes do interior de Mato Grosso ingressaram no serviço de reabilitação com menos de 3 anos de idade, enquanto os de Cuiabá e Várzea Grande, com 3 anos ou mais. Quando um indivíduo nasce ou é diagnosticado com alguma alteração não tratável em seu município, os estabelecimentos de saú de encaminham os casos para o serviço mais próximo de sua residência via Sistema Nacional de Regulação ^26^; assim, os indivíduos com fissuras orofaciais não sindrômicas, no estado de Mato Grosso, são aten didos preferencialmente em Cuiabá ^12^. Isso pode explicar o ingresso de pacientes do interior do estado na idade adequada para o tratamento. No entanto, pacientes de Cuiabá e Várzea Grande que já estavam em acompanhamento em outros estados podem ter escolhido transferir-se para o Serviço de Reabilitação de Fissuras Labiopalatinas do Hospital Geral de Cuiabá após sua implementação, justificando o cadastro dos indivíduos com idade mais avançada e histórico de cirurgias prévias. 

O fato de o paciente residir na zona urbana ou rural não interferiu na idade em que ele ingressa no serviço. Embora a escassez de infraestrutura rural possa atrasar o diagnóstico e o tratamento ^27^, isso não foi iden tificado neste estudo. Três possíveis razões para isso seriam a rápida identificação de malformações faciais, diferentemente de malformações em órgãos internos ^28^; a referência formal no sistema de saúde estadu al para encaminhamento de pacientes com fissuras orofaciais ^26^; e o fato de o Hospital Geral de Cuiabá ser referência estadual para partos de alto risco ^13^, uma vez que há casos de diagnóstico intrauterino de fissura orofacial por meio de ultrassonografias ^22^. Assim, desde 2008, o hospital geral passou a receber, de forma sistematizada, os pacientes nascidos com a deformidade no estado ^13^. 

Observou-se maior ingresso de indivíduos com 3 anos de idade ou mais e histórico de cirurgias nos primeiros períodos; e, nos últimos períodos, prevale ceu o ingresso de pacientes com menos de 3 anos e sem cirurgias prévias. Isso reflete a situação anterior à criação do serviço, quando os pacientes precisavam buscar atendimento em serviços espalhados pelo Brasil, devido à ausência de um serviço especializado em Mato Grosso. Durante esse período, a distância e a falta de acompanhamento dificultavam o tratamento, e muitos pacientes recebiam somente o tratamento cirúrgico e ficavam desassistidos ^12^. Em 1999, a Operation Smile realizou a “Operação Sorriso Brasil” no Centro-Oeste, iniciando por Cuiabá ^29^, deixando vários pacientes sem acompanhamento multiprofissional após as cirur gias. Isso também ajuda a explicar por que os primeiros pacientes cadastrados no serviço de reabilitação já haviam sido submetidos a cirurgias, ao passo que, a partir do segundo período, os pacientes passaram a ingressar, em sua maioria, sem cirurgia prévia, para dar início ao tratamento. 

A mudança no perfil dos ingressantes no Serviço de Reabilitação de Fissuras Labiopalatinas do Hospital Geral de Cuiabá, durante o período estudado, pode ser atribuída à implantação do próprio serviço, que facilitou o acesso ao tratamento e regularizou a demanda local represada. Como resultado, os pacientes começaram a buscar atendimento já nos primeiros anos de vida. A antecipação do início do tratamento também foi observada em Belém, Pará, onde o número de cirurgias realizadas no momento ideal aumentou em 12% entre os primeiros e os últimos quatro anos do serviço ^8^. 

No entanto, um estudo que avaliou os 28 hospitais brasileiros credenciados para o tratamento de fissura orofacial revela disparidades regionais, especialmente nas regiões Norte, Nordeste e Centro-Oeste, que ainda enfrentam uma disponibilidade limitada de serviços ^30^. No caso do estado de Mato Grosso, o tamanho do território e a distância dos municípios em relação à capital podem dificultar o pleno acompanhamento multiprofissional ^10^, já que o tratamento exige con sultas frequentes, ao longo do tempo, para o completo restabelecimento desses indivíduos ^11^. Essa difi culdade no acesso pode comprometer a eficácia do tratamento e o prognóstico dos pacientes. Portanto, sugere-se a realização de novos estudos, a fim de se avaliar a necessidade de implementar outros serviços descentralizados para suprir essa possível demanda; estudos que incluam diferentes centros, a fim de se analisar se o perfil dos pacientes está de acordo com o encontrado; e se os indivíduos do centro estudado com tratamento prévio receberam a intervenção na idade ideal, os locais onde os tratamentos foram realizados e os procedimentos cirúrgicos mais comuns. Sugere-se ainda a inclusão de informações sobre lateralidade (unilateral, bilateral) e os subtipos de fissura (completa, incompleta) nos estudos futuros. 

A análise do perfil dos pacientes ingressantes no Serviço de Reabilitação de Fissuras Labiopalatinas do Hospital Geral de Cuiabá revela predomínio de indi víduos do sexo masculino, com fissura labiopalatina, originários da zona urbana do interior de Mato Grosso e sem histórico de procedimento cirúrgico prévio. O perfil mudou ao longo do tempo, com uma transição de maior ingresso de pacientes mais velhos e com histórico de cirurgia prévia para maior ingresso de pacientes mais jovens e sem cirurgia prévia.
